# Practical utility of liver segmentation methods in clinical surgeries and interventions

**DOI:** 10.1186/s12880-022-00825-2

**Published:** 2022-05-24

**Authors:** Mohammed Yusuf Ansari, Alhusain Abdalla, Mohammed Yaqoob Ansari, Mohammed Ishaq Ansari, Byanne Malluhi, Snigdha Mohanty, Subhashree Mishra, Sudhansu Sekhar Singh, Julien Abinahed, Abdulla Al-Ansari, Shidin Balakrishnan, Sarada Prasad Dakua

**Affiliations:** 1 Surgical Research, Hamad Medical Corporation, Doha, Qatar; 2 Texas A&M University, Doha, Qatar; 3 McGill University, Montreal, Canada; 4 KIIT University, Bhubaneswar, India

**Keywords:** Liver, Tumor, Segmentation, Surgery, Intervention

## Abstract

Clinical imaging (e.g., magnetic resonance imaging and computed tomography) is a crucial adjunct for clinicians, aiding in the diagnosis of diseases and planning of appropriate interventions. This is especially true in malignant conditions such as hepatocellular carcinoma (HCC), where image segmentation (such as accurate delineation of liver and tumor) is the preliminary step taken by the clinicians to optimize diagnosis, staging, and treatment planning and intervention (e.g., transplantation, surgical resection, radiotherapy, PVE, embolization, etc). Thus, segmentation methods could potentially impact the diagnosis and treatment outcomes. This paper comprehensively reviews the literature (during the year 2012–2021) for relevant segmentation methods and proposes a broad categorization based on their clinical utility (i.e., surgical and radiological interventions) in HCC. The categorization is based on the parameters such as precision, accuracy, and automation.

## Introduction

The World Health Organization (WHO) has reported Hepatocellular Carcinoma (HCC) as the leading cause of cancer deaths worldwide. In 2020, liver cancer has resulted in 830,000 deaths, and HCC has accounted for about 80% of primary liver cancers [1]. Surgeons, radiologists, and oncologists study liver physiology, pathology and morphology using multiple tools to evaluate the disease state. For example, imaging modalities such as CT/MRI images are utilized for computer-aided diagnoses (CAD) to evaluate pathologic liver conditions. Segmentation of CT/MRI liver images greatly augment clinical decision support by playing an essential role in existing CAD systems. They enable the surgeons to identify the lesions that have even similar gray-level intensities as the liver, and help them devise case-appropriate treatment pathways. Precision and reliability of segmentation methods are important for obtaining clinically relevant boundary and volumetric assessments in staging of liver tumors (e.g., Response Evaluation Criteria in Solid Tumor (RECIST) protocol) [2].

Accurate delineation of liver and tumor helps in appropriate planning for HCC treatment [3, 4]. Thus, liver and tumor segmentation methods play a critical role in treatment approaches such as Radio-frequency Ablation (RFA), Percutaneous Ethanol Injection (PEI), Selective Radiation Therapy (SIRT), Transcatheter Arterial Chemoembolization (TACE), and the use of targeted agents [5]. Liver and tumor segmentation are also a prerequisite for surgical resection [6]. Segmentation is also crucial in post-interventional tracking of ablated/resected tissues of the liver; it also ensures appropriate negative tissue margins, allowing the clinician to evaluate the efficacy and success of the procedure. Liver and tumor segmentation thus play an important role in the diagnosis, treatment, and follow-up of HCC patients [5].

The categorization of segmentation methods has been often subjective; they are generally classified based on their methodology or extent of human intervention. Methodology based categorizations include model-based (e.g., active contours [7], statistical shape models [8], and graph cuts [9]), or intensity-based (e.g., region growing [10]) approaches. Model-based approaches tend to attain better segmentation performance than intensity-based methods due to their accurate statistical and mathematical modeling for capturing the region of interest. However, model-based approaches may require parameter tuning and initialization, resulting in high computational time. Segmentation methods can also be classified as semi-automatic or automatic based on the extent of human intervention. The semi-automatic methods require the surgeon’s assistance, thereby providing more control to the surgeons during the procedure. On the other hand, the automatic methods have minimal user errors due to a lack of external interventions. However, the automatic segmentation methods are biased towards the statistical distribution of the training data. Recently, deep learning-based (i.e., model-based) methods have gained popularity due to advances in accuracy, robustness, and generalization provided by the neural networks [11–20].

**Fig. 1:**
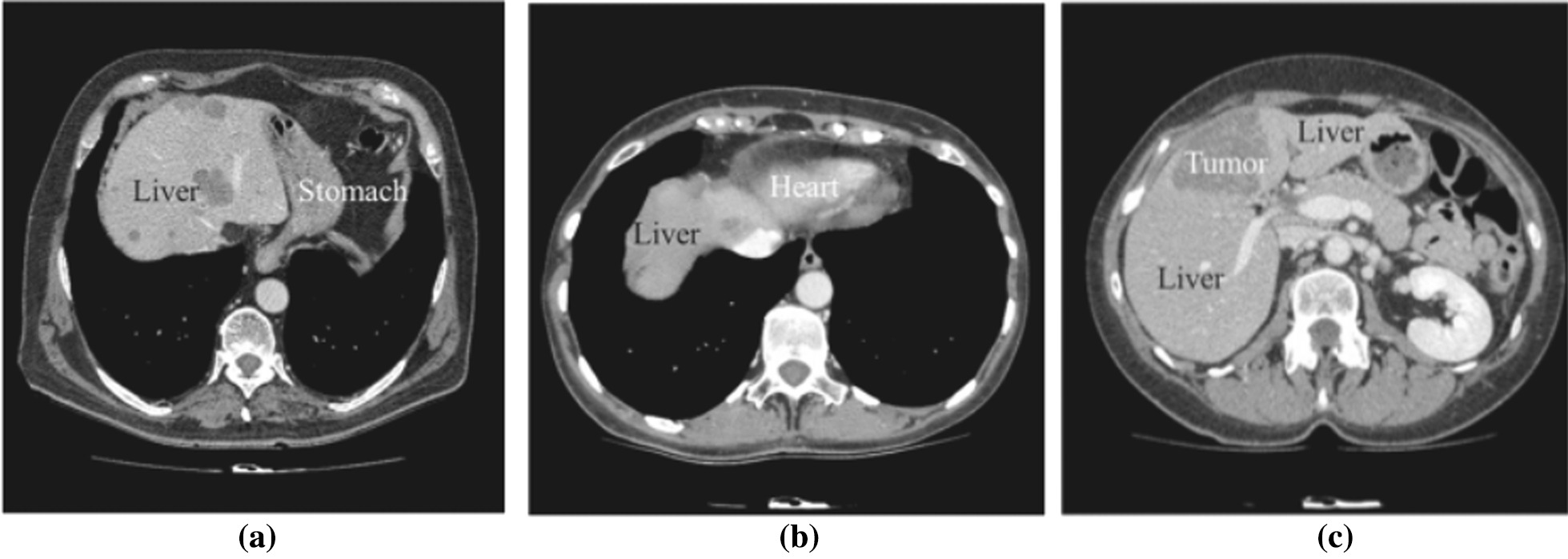
Examples of challenges in liver segmentation: **a** ambiguous boundary between liver and stomach, **b** ambiguous boundary between liver and heart, **c** similar intensity of liver and tumor

Differences in liver morphologies and pathologies between people result in varying liver volumes and shapes in CT/MR imaging, thereby adding complexity to the development of accurate liver and tumor segmentation methods. Furthermore, usage of contrast media injections increases the complexity of the segmentation process, because it alters the grey level values of the liver, making its intensity similar to neighboring tissues/organs such as the stomach, spleen, and abdominal wall [21]. Figure 1 shows the challenge faced by the segmentation algorithms due to ambiguous anatomical liver boundaries. Liver lesion segmentation is also challenging due to variable contrast levels (i.e., hyper-/hypo-intense tumors) and broad-spectrum abnormalities (inconsistent size and shape of lesions) [5].

Thus, in order to suggest utility within surgical hepatobiliary interventions, it would be useful to first compare and categorize different segmentation methods with respect to their accuracy, extent of automation, and segmentation performance. These metrics are expected to vary based on liver pathology and morphology, as well the staging and spread of malignancies such as HCC. Some efforts have already been made in this regard, for instance, Bilic et al. [5] assess over 24 state-of-the-art liver and tumor segmentation methods and conclude that a single segmentation algorithm might not always be the best fit for segmenting the liver and its tumors.

In this paper, we propose a categorization schema of segmentation methods with respect to their utility in diagnosing HCC and planning hepatobiliary surgical interventions. Specifically, we consider suggesting segmentation methods for the outcomes of an early-stage HCC treatment protocol (i.e., BCLC). The diagnosis of HCC is a complex procedure that may be impacted by existing liver morphology (due to diseases such as liver cirrhosis) [22]) as well as tumor pathology (size [23], intensity [24], malignant or benign nature [25]). There have been extensive publications in the literature, which classify and detect tumors [26–28]. In this work, we categorize the existing liver and tumor segmentation methods as per their suitability for appropriate therapeutic pathways. The scope of the paper is to propose a categorization schema that could serve as a knowledge base for the treating physician (surgeon/radiologist) for expediting their segmentation tool selection from existing choices. The primary focus of our paper is on segmentation methods of the liver and tumor. In order to accomplish this, We have extensively reviewed the literature (surgical and nonsurgical interventions for HCC treatment) and categorized them based on some pre-defined critical parameters such as accuracy, automation, and precision.

This paper is structured as follows; Section 2 provides an overview of the liver and tumor segmentation methods. Section 3 and 4 present the segmentation methods for surgical and radiological interventions, respectively. Section 5 discusses the technical and clinical challenges facing the segmentation algorithms and treatment of HCC. Section 6 concludes the paper and provides critical future directions related to the liver and lesion segmentation.

## Literature review

**Fig. 2:**
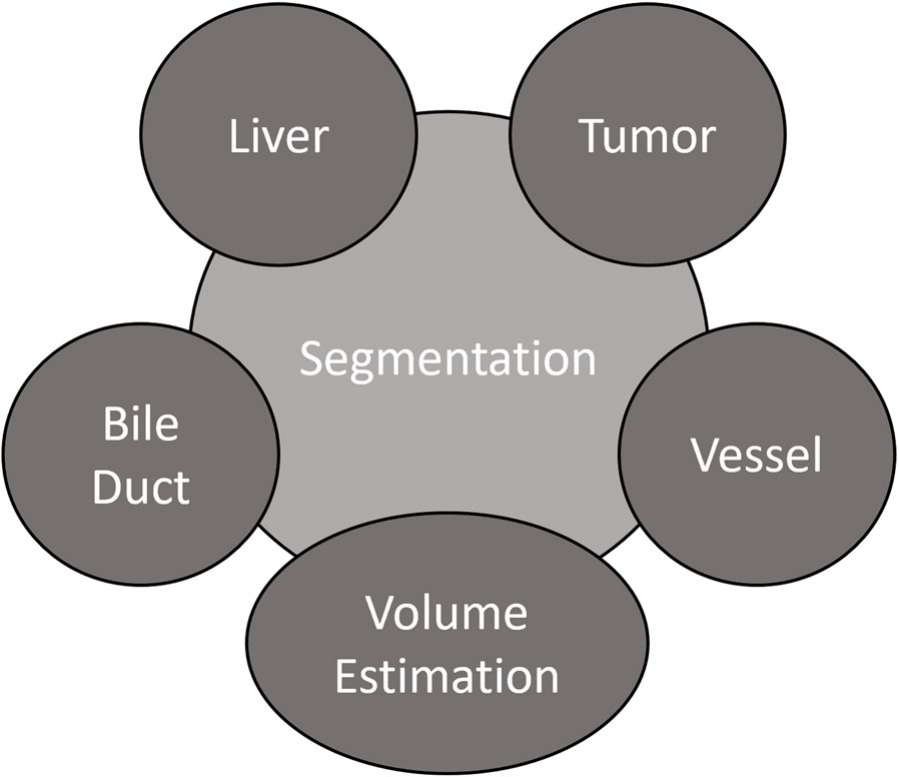
Applications of segmentation methods for liver diseases

Liver segmentation in CT and MRI scans is challenging due to variability in liver dimensions and comparable gray-level intensity of its neighboring organs (e.g., heart and kidney). Furthermore, the blurry anatomical boundaries, poor contrast of the medical images, partial volume effects resulting from patient movement, spatial averaging, and reconstruction artifacts make the liver segmentation daunting. Figure 2 describes the application of segmentation for the other parts of liver.

Generally, the conventional image segmentation methods are either model-based (e.g., active contours, and snake algorithms) [29–33] or intensity-based (e.g., thresholding). Some algorithms utilize primitive image features, e.g., pixels intensities in region-growing/thresholding based approaches [30, 34–36]. Fuzzy segmentation methods have also been utilized for multi-channel image segmentation and extended for single-channel images [37, 38]. Zhang et al. [8], and Nuzillard et al. [39] have shown that model-based statistical approaches achieve expected results relative to the conventional segmentation methods based on image intensities. Mahr et al. [40] evaluate several segmentation methods and conclude that model-based methods are the potential futures for liver segmentation. However, the statistical models are limited by constraints and require additional parameter tuning and initialization, resulting in high computational time. If the models are evaluated without the constraints, they have the potential to misclassify or under-segment the region of interests.

As discussed earlier, there is also a different categorization that is based on the extent of human intervention; some of them are automatic [41–48] that do not require human intervention for the generation of segmentation masks and some are semi-automatic requiring human assistance, say for seed selection or segmentation mask refinement [49–53]. Linguraru et al. [42] suggest an automatic segmentation approach for liver segmentation based on an affine invariant shape formulation. The paper makes a point-to-point comparison of various 3D surface features in the affine parameter space. Another automatic method proposed by Seo et al. [54] follows a multi-stage approach by utilizing an optimal threshold value to segment liver, hepatic vessels, and tumors sequentially. Chartrand et al. [49] introduce a semi-automatic liver segmentation technique that generates an approximate liver model and deforms it by using a Laplacian mesh optimization to obtain accurate liver segmentation. Peng et al. [52] utilize semi-automatic level sets that integrate the likelihood energy and anatomical boundary information to segment the liver. Zhang et al. [50] propose a semi-automatic method based on Couinaud’s theory to segment the liver with varying clinical conditions. Zhao et al. [55] report a semi-automatic region-growing method that avoids over-predictions of the surrounding tissues and organs using shape constraints. The primary shortcoming of semi-automatic implementations is user intervention that interrupts the segmentation process and results in subjective outcomes. From this standpoint, it is essential to note that automated segmentation methods are preferred in time-constrained clinical applications.

Segmentation of vessels, tumors, and bile duct also play an important in the diagnosis, treatment, and post-treatment evaluation of HCC [56, 57]. These liver components are relatively minute, thus their segmentation is certainly challenging. In addition, artifacts, poor contrast, and distortions in the standard image at the native scale/resolution increase the segmentation complexity. Specific denoising and image enhancement methods improve the images overpowering the high noise levels and artifacts (e.g., Wavelet and Ridgelet transform). These methods transform the image to a different domain in order to segregate the image noise [40, 58]. Shang et al. [48] propose an active contour-based method that uses a Gaussian mixture model to segment major liver vessels. Then, a vascular vector field centerline segments thin vessels with lower visibility. Kirbas et al. [59] present a comprehensive review to understand the conventional vessel segmentation algorithms. Wang et al. [60] suggest a model-based algorithm to detect and segment bile duct carcinoma. Similar model-based approaches have been proposed for detecting and segmenting liver malignancies (e.g., HCC) [42, 43].

In recent years, machine learning and deep learning have vigorously gained popularity in medical image segmentation. Specifically, the U-net architecture, proposed for biomedical image segmentation, has been modified for the segmentation of organs in CT/MRI images [61]. Although the deep learning algorithms provide acceptable reliability and accurate results, they require large datasets and dedicated hardware (i.e., GPU). The challenges related to limited data and computation cost have been mitigated using data augmentation and efficient network layers.

Alexey et al. [16] present a hybrid convolutional LSTM architecture that merges time distributed convolutions, bi-directional C-LSTM blocks, and pooling operations to work with partial input volumes. The resultant hybrid network is competitive in terms of computational power, memory consumption, and inference times for liver segmentation. Kavur et al. [12] perform an empirical study to evaluate the accuracy and repeatability of twelve semi-automatic and automatic methods. The authors have exhaustively evaluated the methods with segmentation metrics (e.g., VOE, average symmetrical surface distances, etc.) and compared their results with slice-by-slice evaluations and a scoring system. The results highlight that the automatic deep learning methods outperform the conventional semi-automatic methods on all segmentation metrics. Isensee [13] propose a neural network-based segmentation framework (nnU-Net) that automatically configures preprocessing, network architecture, training, and post-processing for biomedical image segmentation. The results indicate that the framework achieves state-of-the-art accuracy on many image segmentation tasks. Furthermore, the nnU-Net framework provides more robust baselines for abdominal organs and sub-structures (e.g., liver, tumor, and vessels). Following the nnU-Net, researchers have organized challenges that improve the practicality and usability of the segmentation methods. FLARE 21^1^ encourages the development of abdominal organ segmentation architectures for CT scans that can be deployed in memory-limited and time-constrained environments. Zhang et al. [14] introduce an efficient context-aware architecture for meeting the objectives of the FLARE 21 challenge, thereby maximizing segmentation accuracy while minimizing inference time and GPU memory consumption. Complementary to FLARE 21, the CHAOS [15] challenge promotes deep learning architectures that generalize well for abdominal organs (e.g., liver) across imaging modalities (i.e., CT and MRI). Conze et al. [62] suggest a conditional generative adversarial network with a partially pre-trained generator to achieve high segmentation accuracy across CT and MRI imaging modalities in the CHAOS challenge. Organ-focused challenges such as KiTS^2^ have been organized to overcome the pathological and imaging modality constraints associated with an organ (e.g., kidney). Chen et al. achieve high accuracy in KiTS 21 by employing a coarse to fine segmentation approach inspired by the nnU-Net framework with the surface loss function to maximize area overlap and minimize surface discrepancies. Altogether, there has been a rapid development of neural network-based solutions for abdominal organ segmentation. In this paper, we aim to suggest a few of the robust liver and tumor segmentation methods to assist in the outcomes (e.g., transplant, resection, and ablation) of the clinical protocols for HCC (e.g., Barcelona staging classification). Table 1 provides a summary of community-organized challenges and some of their publications.

**Table 1:** Recent biomedical segmentation challenges and some of their publications

Challenge	Reference	Dataset	Method	Performance (best results)
CHAOS	Conze et al. [62]	80 patients (40 CT, 40 MRI scans)	Conditional generative adversarial network with a partially pre-trained generator	Dice: 97.95 ± 0.27 ASSD: 0.76 ± 0.16 (performance of cGv16pUNet1-1)
FLARE	Zhang et al. [14]	511 CT scans and annotations for 4 abdominal organs	Context-aware efficient encoder-decoder model with anisotropic pyramid pooling	Dice: 96.5 ± 6.1 NSD: 87.8 ± 11.2 (performance of efficientSegNet)
KiTS	Chen et al. [63]	300 CT scans	nnU-Net-based coarse-to-fine segmentation framework	Dice: 90.99 NSD: 83.48

## Segmentation for surgical intervention

**Fig. 3:**
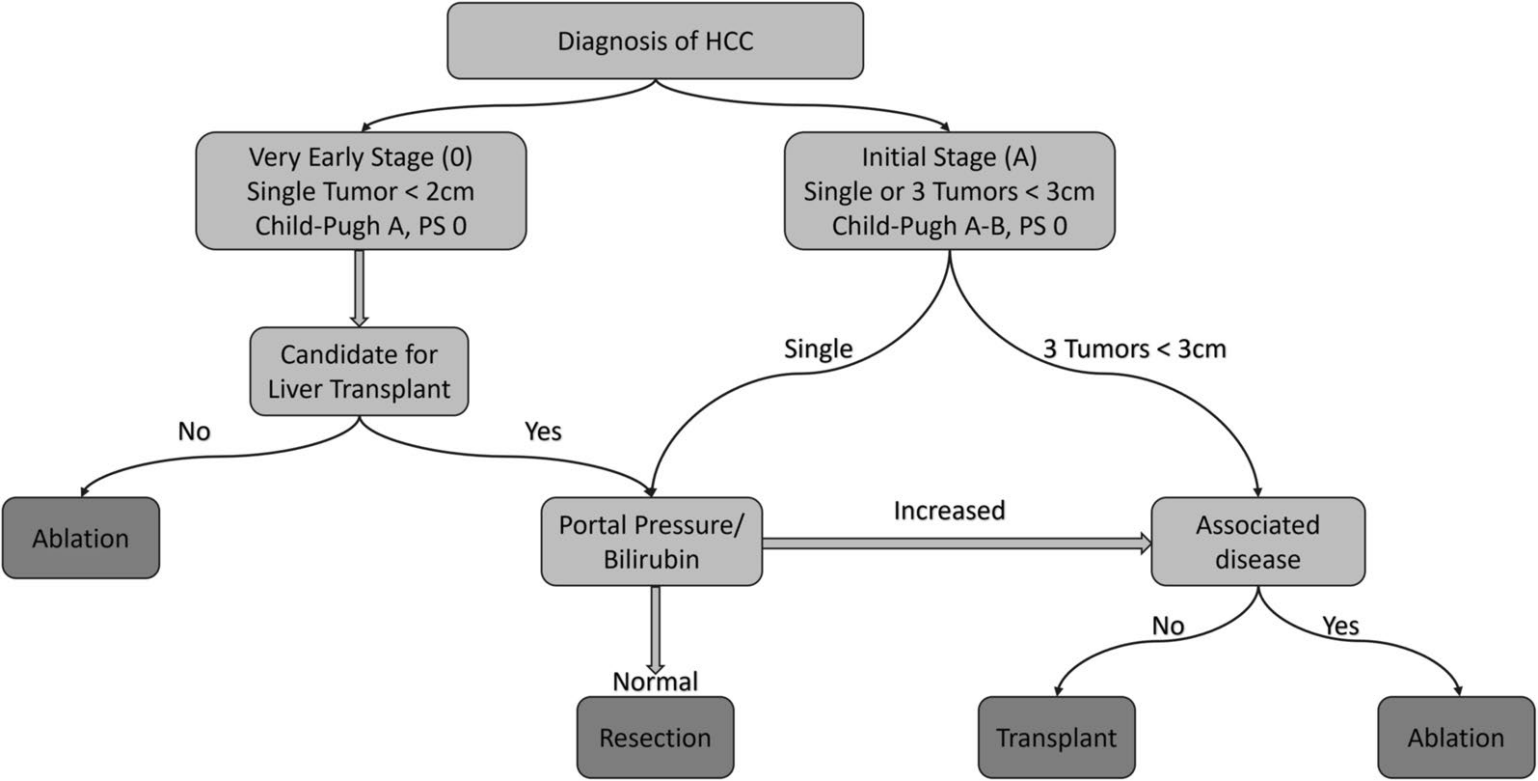
Staging classification and treatment algorithm of very early (0) and early (A) stage HCC based on BCLC criteria

**Fig. 4:**
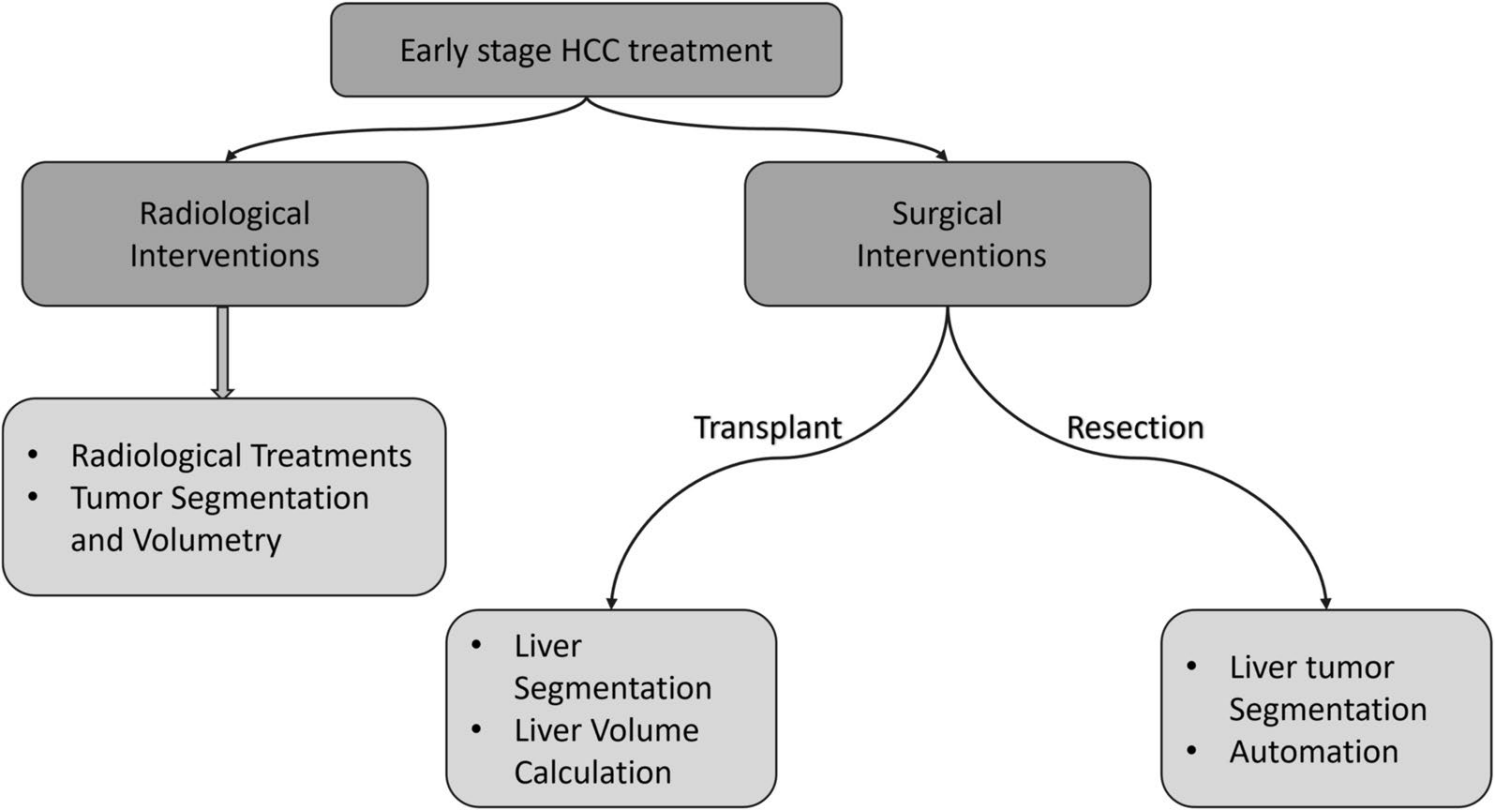
Structural summary of section 3 and 4, highlighting the essential functionalities of segmentation methods for radiological and surgical interventions

HCC prognosis is dependant on tumor stage as well as residual hepatic dysfunction due to cirrhosis. Extent of symptomatic presentation and comorbidities also contribute to prognosis. Various HCC staging systems are used to clinically guide HCC treatment, such as Okuda system, Tumor, Node, Metastasis (TNM) staging, Cancer of the Liver Italian Program (CLIP) score, Barcelona staging classification (BCLC), Albumin-Bilirubin (ALBI) score, etc. [64–68]. Among these staging systems, BCLC system is preferably used by clinicians due to its holistic approach, as it accounts for the extent of hepatic lesion, vascular invasion, hepatic function status, and spread outside the liver [68]. Furthermore, several studies have also reported that the BCLC system outperforms other systems in predicting HCC prognosis due to a more holistic tumor staging [69, 70]. Thus, using BCLC, clinicians are likely to decide the most appropriate therapeutic intervention for patients suffering from HCC. Figure 3 showcases the staging and treatment recommendations according to the BCLC criteria. Studies have shown that patients diagnosed with large multifocal tumors or advanced stage HCC are less likely to benefit from transplantation, liver resection, and ablation therapy [71–74]. Nevertheless, the patients diagnosed with the early or initial stage of HCC with no liver diseases can be treated by surgical liver resection [75]. Figure 4 summarizes the role of segmentation and volumetry in surgical and radiological interventions of the liver.

### Transplant

A liver transplant is recommended for treating patients with very early stages of HCC and increased portal pressure or bilirubin levels 3. However, clinicians need to ensure that the transplanted liver is well matched as this may impact the functional capabilities of the liver. In liver transplant surgeries, accurate segmentation and calculation of liver volume are critical, as the success of the donor and recipient operations depends heavily on the graft size [76]. The accepted standard for liver segmentation and volume calculation is manual delineation, which requires a highly experienced surgeon to manually trace the boundaries of the liver on CT/MRI images. However, manual tracing of organs is time-consuming and idiosyncratic. Therefore, reliable and automated segmentation methods with fast inference times can reduce the complexity of the liver transplantation procedure. It has been shown that structure-based, machine learning, and deep learning methods are suitable for liver delineation and volume estimation because of their robustness and ability to achieve high segmentation accuracy [77, 78]. These methods learn to identify variations of the liver shapes that may be missed by conventional segmentation algorithms, allowing for robust and consistent boundary delineation and volume calculation.

#### Liver segmentation

Liver segmentation in 3-D medical scans is a crucial prerequisite for the calculation of liver volume and liver/tumor ratio (i.e., tumor burden) [84, 85]. Liver segmentation methods with high precision and accuracy (comparable to manual delineation) are highly desirable in clinical workflows. As discussed earlier, the deep learning-based approaches have recently provided robust and accurate liver segmentation methods that may assist in liver transplantation. Alirr et al. [81] employ a region-based level set function with convolutional networks for liver and tumor segmentation. The FCN architecture has been tested on the IRCAD and LiTS dataset and resulted in 95.2%, 95.6% Dice coefficient on the liver, and 76.1 %, 70% Dice coefficient on the tumor, respectively. Yasaka et al. [82] introduce a CNN model to differentiate between liver masses during dynamic contrast agent–enhanced CT. The model is trained using 55,536 image sets (from 460 patients) to learn accurate and precise differentiation between liver regions. Results indicate that the median accuracy is above 0.84 for differential diagnosis of liver masses on the test dataset. Vorontsov et al. [83] propose an FCN architecture for detecting and segmenting liver lesions in CT images for patients with Colorectal Liver Metastases (CLMs). Results show that the network produces high Dice coefficients for increasing lesion size. Specifically, the FCN achieves Dice Similarity Coefficient (DSC) of 0.14 (size<10 mm), 0.53 (size 10-20 mm), and 0.68 (size>20 mm). Altogether, these state-of-the-art methods could serve as an effective second opinion for interventional radiologists responsible for delineating livers in medical scans.

#### Calculation of liver volume

Liver and lesion volumetry, provide valuable information to the surgeons contributing to the success of liver transplantation [5, 86]. Over the years, several automated segmentation methods have been proposed to segment the liver in CT and MRI imaging. Recently, Lu et al. [78] introduce a 3D convolutional neural network (CNN) with graph cut to delineate the liver and predict its volume. The model’s evaluation on the MICCAI-Sliver07 and 3DIRCADb datasets result in a volumetric overlap error (VOE) of 5.9% and 9.36%, respectively. Wang et al. [79] present a computationally light 2D U-Net variant for liver segmentation and volumetry. The model is trained using 330 abdominal CT examinations in two stages, allowing coarse fine segmentation. Results show that the proposed model reaches over 95% agreement with the ground truth. In [80], a comparison of Vivo hepatic automated volumetry with manual volumetry is performed to assess the effectiveness and margin of error for automated segmentation methods in liver transplantation scenarios. These neural network-based systems could provide viable information to the clinicians for deciding donor-patient compatibility based on liver volume estimation. Table 2 provides a summary of liver segmentation and volume estimation methods along with the datasets and performance.

**Table 2:** Summary of methods for liver segmentation and volume estimation

References	Method	Dataset	Performance
Lu et al. [78]	3D-CNN employed for liver detection and probabilistic segmentation, followed by a Graphcut for segmentation refinement.	MICCAI-Sliver07, 3DIRCADB	VOE: 5.9, 9.36 RVD: 2.7%, 0.97% ASD: 0.91, 1.89 RMSD: 1.88, 4.15 MSD: 18.94 mm, 33.14 mm
Wang et al. [79]	2D U-Net trained in two stages to demonstrate the feasibility of transfer learning for CT segmentation	Custom Dataset (330 abdominal MRI and CT scans)	Dice: 0.94 ± 0.06 (CT) Dice: 0.95 ± 0.03 (T1-weighted MRI) Dice: 0.92 ± 0.05 (T2*-weighted MRI)
Nakayama et al. [80]	In vivo comparison of automatic and manual volumetry for liver volume calculation	Custom Volumetric Dataset	Automatic: 982.99 cm^3^ ± 301.98 (volume), 4.4 minutes ± 1.9 (time) Manual: 937.10 cm^3^ ± 301.31 (volume), 32.8 minutes ± 6.9 (time)
Allir et al. [81]	FCN used for coarse liver segmentation, followed by the use of region-based level set function for tumor segmentation	LiTs, IRCAD	Liver Dice: 95.2%, 95.6% Liver Tumor Dice: 76.1%, 70%
Yasaka et al. [82]	Custom CNN architecture for clinical retrospective study on different phases of CT scans	Custom Dataset (55536 Pictures)	Median Accuracy: 0.84 Median AUROC: 0.92
Vorontsov et al. [83]	FCN with two stages forliver and tumor segmentation	Custom Dataset (156 contrast material-enhanced CT scans)	Tumor Dice: 0.14 (size < 10 mm), 0.53 (size 10–20 mm), 0.68 (size > 20 mm)

### Resection

#### Importance of segmentation in resection

**Fig. 5:**
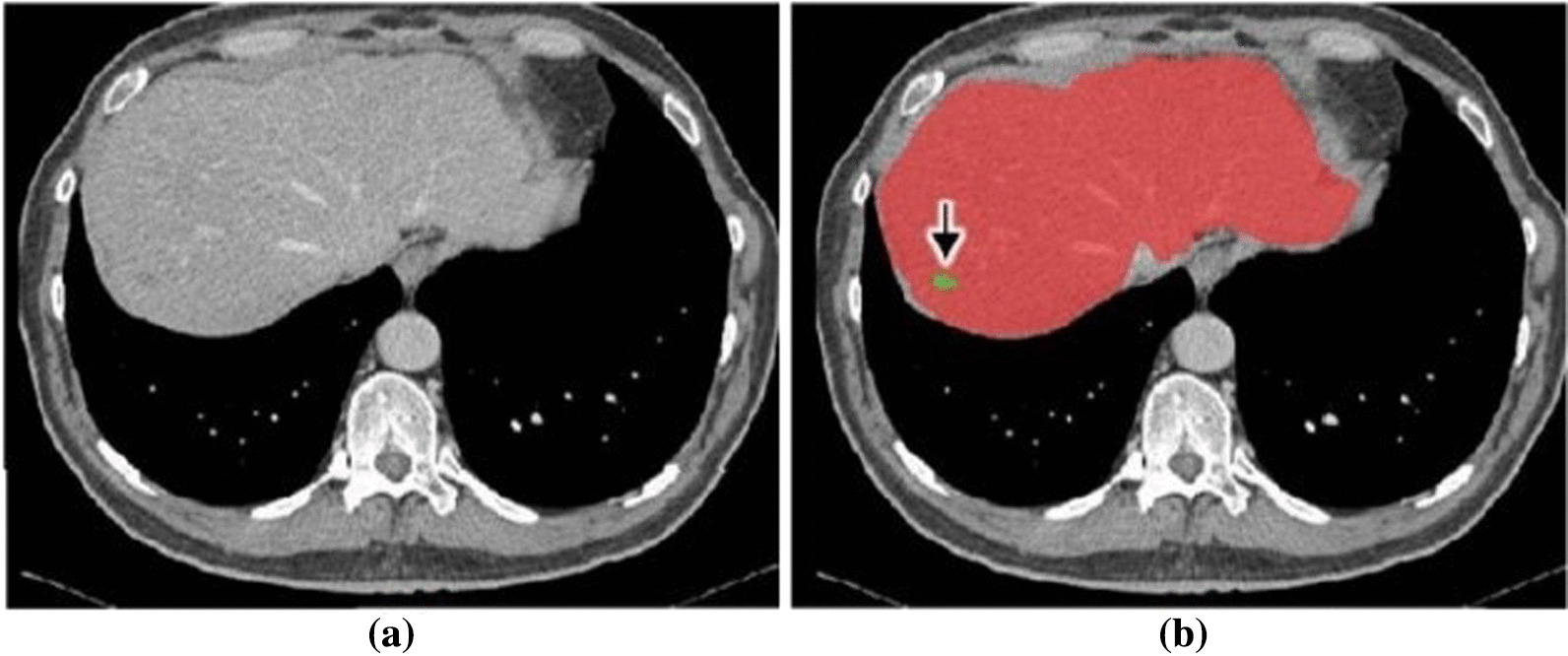
**a** Raw CT slice and **b** Segmented liver

BCLC staging system recommends liver resection for patients suffering from early or initial stage HCC with a single tumor, normal portal pressure, and bilirubin levels. For physicians, tumor information plays a critical role in surgical planning and image-guided interventions. Specifically, the exact volume, morphology, shape, and location of tumors must be accurately determined to carry out a successful resection procedure. In a conventional setting, surgeons manually delineate liver lesions by relying on their experience and observations, which results in biased outcomes that lack efficiency and robustness. Therefore, automated liver tumor segmentation methods are considered as a crucial second-opinion for interventional radiologists and surgeons. However, performing automatic tumor segmentation is quite challenging due to the low intensity, poor contrast, and anatomical variation of liver and lesions between patients. Specifically, sometimes the tumors vary in shape, size, and location, making the algorithm challenging to generalize for a diverse patient population. In addition, unclear boundaries of some lesions (as in Fig. 5) make it difficult for edge-based algorithms to perform effectively. Furthermore, the variations in anisotropic dimensions of the medical scans (i.e., voxel space ranging from 0.45 mm to 6.0 mm) may cause loss of critical volumetric information. Nevertheless, several conventional methods, deformable models, and neural networks have been proposed to segment liver lesions [87–89].

#### Liver tumor segmentation

One significant challenge for the tumor segmentation algorithm is the inconsistency in tumor shapes and locations between patients. Deep learning models can overcome this challenge by using training data that contains a diverse patient population with tumors of different shapes, sizes, and locations. It has been shown in literature that neural networks’ robustness and generalization capability increase with the quantity and diversity of the dataset. There have been a few high-accuracy liver tumor segmentation models based on deep learning: Zhang et al. [88] propose a level-set technique for CT-based liver tumor segmentation that incorporates an edge indicator and an automatically computed initial curve. The method employs a 2D-slice-based U-net to localize the liver, followed by a 3D patch-based FCN to refine the liver segmentation and locate the tumor. The model’s evaluation of the MICCAI 2017 Liver Tumor Segmentation (LiTS) Challenge has resulted in an average DSC of 96.31%. Xi et al. [89] present two cascading U-ResNets for end-to-end liver and lesion segmentation. Results on the LiTS highlight that the model achieves a Dice score of 94.9%. Bai et al. [90] utilize a multi-scale candidate generation method (MCG) and 3D fractal residual network (3D FRN) for liver and tumor segmentation in CT volumes. Initially, a U-Net segments the liver region in 3-D space. Then, MCG is employed to mark the candidate regions for liver tumors. Finally, 3D FRN is used to mark the lesion accurately. It is known that post-processing removes minor mispredictions, enhances/refines segmentation masks, enhancing the generalization capabilities of the deep learning model. Bai et al. [90] use an active contour model (ACM) on the tumor predictions to refine tumor boundaries. The resultant method 3D MCG-FRN + ACM results in a Dice coefficient of 0.67 on the 3DIRCADb dataset. Alternatively, Li et al. [51] propose an approach combining a level set model with likelihood and boundary energies to segment liver tumors. The result highlights a Jaccard distance error of 14.4 ± 5.3% and a relative volume difference of -8.1 ± 2.1% on a custom CT dataset with 18 patients. Recently, Dong et al. [91] propose a Hybridized Fully Convolutional Neural Network (HFCNN) to detect cancer and segment liver tumors.

After segmenting the existing tumors in the liver, the surgeons and radiologists need to recognize its type to determine the extent of cancer spread and malignancy. This classification process can be automated and embedded within the segmentation algorithm, efficiently providing physicians valuable secondary information. Trivizakis et al. [26] train a 3-D CNN using 130 DW-MRI scans to classify the tumor type. The network results in an $83\%$ classification accuracy as compared to $69.6\%$ of a previously implemented 2-D CNN. Chen et al. [27] present a probabilistic neural network (PNN) that is trained using fractal information gray-level co-occurrence matrix to classify liver tumors into hepatoma and hemangioma. Balagourouchetty et al. [28] suggest an ensemble FCNet classifier trained using GoogLeNet features to classify six different classes of liver tumors.

#### Automation

The heterogeneous shape of tumors, and inconsistent background, creates high unpredictability between the liver and the lesions, thereby adding complexity to automatic tumor segmentation methods [77]. Most interactive or semi-automatic methods that involve input from a physician have shown better results and are used for critical hepatic operations like hepatic biopsies and hepatic therapeutic interventions [96]. Zhang et al. [97] present an interactive seed-selection strategy for liver tumor segmentation using support vector machines in CT scans. Lin et al. [92] propose an interactive implementation that places emphasis on region partition and boundary information. The tumor texture information and clear tumor boundary allow the model to segment tumors effectively. Moreover, the Lucas–Kanade algorithm selects the seed pixel for initiating model training, and user inputs are utilized to incorporate the data variations. The collaborative model obtains promising results and an average segmentation accuracy of 80%. On the other hand, fully automated methods lack performance because of the complex and volatile nature of surgeries and complications [98]. Nonetheless, complete automation is being consistently pursued to achieve performance that is comparable to semi-automatic methods. A fully automated deep learning approach based on Attention Hybrid Connection (AHC) Network architecture is implemented by [93], giving decent results. The network is tested using 20 cases from the 3DIRCADb dataset and 117 cases from a clinical dataset, achieving a global Dice coefficient of $0.62 \pm 0.07$ in tumor segmentation. Seo et al. [94] proposes a modified U-Net (mU-Net), which combines object-dependent high-level features to improve liver-tumor and liver segmentation from CT scans. The model’s evaluation on the (LiTS) dataset results in a Dice similarity coefficient (DSC) of 89.72 % for liver tumors. Vivanti et al. [95] present an automatic method for liver tumors segmentation in post-treatment CT studies that use a CNN to image patches. Next, a voxel classifier is employed to generate the refined tumor segmentation mask. The model’s evaluation on a custom dataset results in an average of 16.05% VOE and a 2.05 mm average symmetric surface distance (ASSD), giving a success rate of 90.5%. Table 3 provides a summary of liver tumor segmentation methods along with the datasets and performance.

**Table 3:** Summary of available methods for liver tumor segmentation

Reference	Method	Dataset	Performance
Lin et al. [92]	Lucas-Kanade algorithm is used for discriminative training, followed by inference algorithm, which employs Lagrangian method and image sequence matching	LiTs	Accuracy: 0.8561 (SYSU-CT), 0.6571 (SYSU-US)
et al. [88]	2D-Slice Based U-Net and 3D Patch-Based CNN are employed for segmentation of liver and localization of tumor. Level-set method is used for tumor refinement	LiTs	Liver Dice: 96.31% ± 0.62% Liver RMSD: 1.99 mm ± 0.64 mm Tumor Dice: 72.45% ± 13.42% Tumor RMSD: 4.99 mm ± 2.18 mm
Xi et al. [89]	Two Cascading U-ResNets for liver and tumor segmentation with a experimental study for measuring the impact of loss functions	LiTs	Liver Dice: 94.9% Liver VOE: 0.0095 Tumor Dice: 75.2% Tumor VOE: 0.379
Jiang et al. [93]	Cascaded Attention Hybrid Connection Network with a combination of soft and hard attention for liver and tumor segmentation	Training set: LiTS Test set: 3DIRCADb (20 patients), Clinical Dataset (117 cases)	0.62 ± 0.07 (DSC)
Seo et al. [94]	Modified U-Net (mU-Net) architecture with the residual path deconvolution over the skip-connections to prevent duplication of low-resolution information	LiTs	Liver Dice: 98.51% Liver VOE: 3.07% Tumor Dice: 89.72% Tumor VOE: 21.93%
Vivanti et al. [95]	CNN trained with delineation of baseline CT scans and evaluated on follow up CT studies	Custom Dataset (67 Tumor in 21 scans)	VOE: 16.26%
Bai et al. [90]	Multi-scale candidate generation method (MCG), 3D fractal residual network (3D FRN), and active contour model (ACM) are used in a coarse-to-fine manner for liver tumor segmentation	Training set: LiTS Test set: 3DIRCADb	Tumor Dice: 0.67 Tumor VOE: 0.324 Tumor MSD: 7.113 mm

## Segmentation for radiological intervention

Interventional radiology has opened new avenues for the treatment of liver cancers. BCLC staging system recommends radiological interventions (e.g., ablation) for patients not suited for transplant or with livers with associated diseases. Radiological treatments can be performed by an endovascular approach or by direct transcapsular access [99]. Endovascular treatments include TACE, Stereotactic Body Radiation Therapy (SBRT), Transarterial Radioembolization (TARE), and portal vein embolization (PVE). Direct transcapsular access treatments involve microwave thermal ablation (MWA), RFA, and PEI [100].

TARE and TACE block the hepatic artery to treat the liver cancer segment by cutting off its blood supply. TARE is a selective internal radiation therapy that requires an intra-arterial supply of microspheres packed with radioactive compounds such as ttrium90, iodine131, or rhenium188 [101]. In comparison, TACE is a type of chemoembolization that involves chemotherapy. CT or MRI imaging is used to predict whether or not extra-hepatic arteries augment tumors. All of the feeding arteries of a tumor, including any possible extra-hepatic arteries, are examined by angiographic images. TACE can treat liver tumors larger than 5 cm, but it may take 2 or 3 treatments [102]. Furthermore, CT scans must be taken 2 to 3 months after TACE to ensure treatment success [103]. Liver tumor segmentation methods may provide crucial secondary information to monitor treatment progress and success.

PVE increases the volume of the Future Liver Remnant (FLR) for extended hepatectomy by embolizing a portal vein region, resulting in hepatic regeneration. PVE is performed, when a large FLR is required for a post-operative liver recovery as determined by liver volumetry. Often this is due to the extent of the liver resection or the underlying liver disease [104]. Segmentation and volumetry of CT scans provide crucial pre-requisite information for the success of PVE. SBRT has been used in the treatment of primary HCC (with slight metastases) that require radiation in less than 25% [105].

RFA and MWA use image guidance intervention, where a probe is utilized for heat generation, resulting in coagulation necrosis to destroy the cancer cells [99]. PEI is performed for tumors less than or equal to 3 cm. PEI injects highly concentrated alcohol using a thin needle, leading to complete ablation of up to 70% of lesions. Ultrasound guidance is generally utilized for performing ablation, and the treatment requires 4-6 sessions. Real-time segmentation of the captured images can assist the radiologist in carrying out the procedure and improve treatment success.

### Tumor segmentation and burden estimation

FLR, Total Liver Volume (TLV), and liver burden are all important volumetry metrics needed for radiological intervention treatment planning [106]. CT or MRI segmentation and volumetry can provide crucial secondary information for radiologists to carry out radiological interventions. Advancements in radiation therapy procedures and segmentation technology effectively reduce GI toxicity (i.e., toxicity in small intestine and stomach) and spinal cord toxicity caused by inadequate/inaccurate dosage determination, leading to more liver dysfunction. For example, radiation-induced liver disease can result from inadequate treatment SBRT planning [107]. Having an accurate liver tumor segmentation enables the secure computation of chemical and radio dosages. Therefore, accuracy and precision are essential considerations for segmentation methods for these applications.

Deep-learning approaches that involve cascaded U-Net derived architectures are accurate with respect to performance [5]. According to the literature, the top three ranked methods according to tumor burden estimation (quantified by RMSE) include Li et al.’s [108] Adaboost to identify tumor boundaries, Wu et al.’s [9] supervoxel-based graph cuts, and Wang et al.’s [109] adaptive mesh expansion model (AMEM) for segmentation of liver. These methods accurately predict the tumor volume with a root mean square error (RMSE) in tumor burden of 0.0150, 0.0160, and 0.0160, respectively [5]. In general, these methods also achieve good area-overlap with the ground truth with Dice scores of 0.9650, 0.9590, and 0.962, respectively [5]. Yuan et al. [110] have also proposed a hierarchical CDNN that performs coarse to fine liver, tumor segmentation, and tumor burden analysis. Despite the state-of-the-art performance, automatically segmenting small liver tumors remains a difficult task. This limitation suggests that future improvements can be made by investigating methods that segment a broad spectrum of liver tumors. Table 4 summarizes the methods, datasets, and performance metrics, for radiation therapy of the liver.

**Table 4:** Segmentation methods for radiation therapy (RT)

Reference	Method	Dataset	Performance
Li et al. [108]	Voxel-based Adaboost is used for liver localization. Shape and appearance models are employed to segment the liver, followed by free form deformation for refinement	MICCAI Sliver07	Liver Dice: 0.911 ± 0.010 (CT), 0.922 ± 0.011 (CTce) Tumor burden RMSE: 0.015
Wu et al. [9]	Liver volume is extracted by histogram-based adaptive thresholding and morphological operations, followed by graph cuts	MICCAI Sliver07	VOE: 7.54% RVD: 4.16% ASD: 0.95 mm RMSD: 1.94 mm MaxD: 18.48 mm Run time: 12.21 sec Tumor burden RMSE: 0.016
Wang et al. [109]	Adaptive mesh expansion model (AMEM) is used for liver segmentation from CT scans. A virtual deformable simplex model (DSM) is introduced to represent the mesh	MICCAI Sliver07	Mean overlap error: 6.8% Mean volume difference: 2.7% ASSD: 1.3 mm RMSD: 2.7 mm Tumor burden RMSE: 0.016
Yuan et al. [110]	Hierarchical convolutional-deconvolutional neural networks (CDNN) for liver and tumor segmentation, followed tumor estimation	LiTS	Liver Dice: 0.967 Liver RMSD: 2.303 Tu mor Dice: 0.82 Tumor RMSD: 1.678 Tumor burden RMSE: 0.017

## Discussion

The diagnosis and treatment planning of hepatic diseases (like HCC) are generally decided by the location and spread of liver lesions, proximity to the vasculature, severity of underlying liver dysfunction, availability of medical technology, and expertise of the clinician. Thus, the choice of segmentation methods for a particular intervention is determined by the method’s robustness, segmentation accuracy, precision, and the extent of automation. For this reason, we have categorized the popular segmentation methods based on their utility. The categorization allows the clinicians to select appropriate methods for the most effective treatment pathways, subsequently aiding clinical decision making in complex diagnostic protocols, and in treatment planning. As a result, it is meant to be a supplementary aid aimed to improve clinical outcomes. Based on the BCLC staging classification (Figs. 3, 4), we suggest segmentation methods for liver transplantation, resection, and radiological intervention. Next, we sub-classify the papers based on their aim to address automation, volume estimation, and segmentation. The models presented by Alirr et al. [81] and Vorontsov et al. [83] use FCNs to achieve high accuracy and precision results for liver segmentation. Wang et al. [79] propose a two-dimensional U-Net CNN to estimate liver volumes in CT scans. Vivanti et al. [95] present an automatic method for liver and tumors segmentation using deep CNNs.

**Fig. 6:**
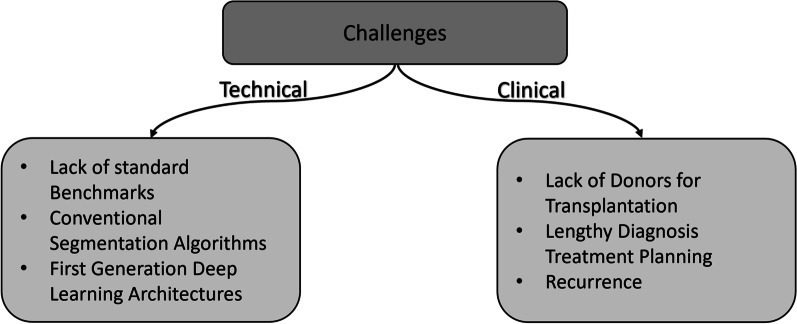
Technical and clinical challenges facing diagnosis and treatment of HCC

Over the past few decades, a significant number of articles have been published to assist the treatment of hepatic diseases (e.g., liver cancer) by proposing different methods for liver and tumor segmentation in CT and MRI scans. However, medical image segmentation of the liver still faces unaddressed challenges in technical and clinical settings. Figure 6 summarizes the clinical and technical challenges for HCC and liver segmentation algorithms, respectively. Some of the technical challenges are lack of standard benchmarks, reliance on conventional segmentation methods, and usage of first-generation deep learning architectures. Similarly, clinical challenges include shortage of compatible liver donors, lengthy diagnosis and treatment planning periods, limited understanding of tumor shapes and morphology.

### Outlook on technical challenges

Comparative studies are critical for discovering state-of-the-art treatment methods for any disease. Many such studies aim to determine the ideal technique for liver segmentation but rely on private, or custom datasets [79, 80, 82, 83, 95]. The datasets often have built-in biases (e.g., subjects diagnosed with a particular disease) and do not cover more than a few hundred patients. Furthermore, the comparison of these works in literature is challenging due to their differing performance metrics. This variability in research due to custom datasets and varying performance metrics add to the challenge of evaluating different methods based on their claimed results, reinforcing the need to create benchmark datasets.

Benchmark datasets and their evaluation using standard metrics are crucial for fair quantitative comparison of the existing methods. It should be ensured that the datasets satisfy both technical and clinical standards. A reliable technical benchmark should contain samples from many patients (with minimal bias) and several slices per scan for each patient. From a clinical standpoint, multiple experts should medically validate and annotate datasets to account for subjective annotation. These guidelines would allow the creation of well-rounded datasets that could aid both technical and clinical challenges facing liver and tumor segmentation.

Robust and accurate segmentation methods are a necessity for clinicians performing liver transplants or resections [111]. After reviewing several works, we have observed that the conventional methods and first-generation neural networks (like plain U-nets and FCNs) have undesirable sensitivity to segment the liver and its tumors, high computation time, and over-segmentation. There has been a rapid development of neural network-based frameworks such as the nnU-Net [13] that automatically configures preprocessing, network architecture, training, and post-processing for biomedical image segmentation, providing good out-of-the-box performance. However, the recent challenges (Like FLARE 21, KiTS 21, and CHAOS [15]) for abdominal organ segmentation of CT and MRI scans have shown that custom CNN architectures [14] with dedicated modules designed for overcoming the pathological challenges outperform the nnU-Net framework. These shortcomings highlight the need for sophisticated deep learning models and dedicated network modules with optimizations to help reduce the networks’ parameter count, memory footprint, and computation time. To improve the current state-of-the-art methods, we propose exploring three avenues in supervised deep learning. First, we encourage employing transfer learning and larger benchmark datasets to tackle overfitting in neural networks. Second, using preprocessing on the datasets to enhance anatomical boundaries and contrast of the images. The preprocessing in CT images is critical due to image noise, poor contrast, and organs with overlapping boundaries. Researchers have utilized conventional denoising algorithms for enhancing CT images. However, new deep learning-based architectures that could simultaneously denoise and segment CT images will be an asset for clinicians to diagnose and treat HCC. Third, we suggest using standard baselines and recent models for comparison (e.g., nnU-Net), providing the community with meaningful results.

Evaluation metrics quantify the effectiveness of segmentation methods for different clinical scenarios. Volume-based metrics (i.e., DSC, IoU, VOE) and boundary-based metrics (i.e., Hausdorff distance (HD), ASSD, etc.) are used to evaluate the segmentation methods. Reinke et al. [112] summarize the pitfalls of the existing segmentation metrics by providing several ground-truth and prediction scenarios where an undesired prediction may receive an acceptable metric score. The authors highlight that DSC has high sensitivity when the target region of interest (ROI) has a size comparable to the pixel size. A similar sensitivity is observed for HD when the resolution of the ground truth varies. The authors highlight that a higher HD is obtained for low-resolution ROI. These findings are crucial for liver tumor segmentation in low-resolution CT images where the tumor is few pixels wide. The authors also show that over-segmentation results in higher DSC for a reference ground truth than under segmentation. Thus, a segmentation method trained to maximize DSC for a liver tumor may tend to over-segment, thereby increasing the chances of resection/ablation of healthy tissues during surgeries. Based on the above findings, we suggest to develop a more robust evaluation metric that effectively quantifies volume and boundary overlap across different ROI sizes/resolutions.

Visualization tool functionalities and user interface may also impact the utility of the segmentation methods in clinical surgeries. Fischer et al. [113] propose a 3D presentation state (3DPR) for parameterizing and storing 3D images. The authors show that 3DPR minimizes user interactions and provides a storage efficient representation for 3D image visualization. One limitation of the approach is that it doesn’t store the segmented 3D image data within the 3DPR object, requiring clinicians to deal with the 3D scan and its segmentation mask separately. Fischer et al. [114] also propose a system that incorporates multiple segmentation methods as plugins and renders the output as a single 3D image. This approach is clinically meaningful, because segmentation masks of different anatomical structures allow radiologists to understand the relationships between the neighboring organs and vessel trees. However, the system doesn’t provide an option to manipulate and remove specific structures from the combined segmentation masks. This functionality is crucial, when the segmentation methods over/under-segments the target anatomical ROI. We recommend developing a visualization system that can incorporate the output of multiple segmentation methods, while providing functionalities to edit the combined segmentation mask. The ability to amend the segmentation masks can allow the radiologist to generate complex annotations for medical image analysis.

### Outlook on clinical challenges

RFA has shown promising results in treating HCC and metastatic diseases such as colorectal cancer (CRC) [115]. Though surgical resection is the gold standard, RFA plays a crucial role in treating patients with inadequate residual liver functionality, multi-lobar lesions, extra-hepatic disease, proximity to prominent vascular structures and co-morbidities, making the patients ineligible for surgery, [116, 117]. However, RFA is associated with vascular and visceral damage, hemorrhagic complications, infections, biliary tract damage, liver failure, and local tumor relapse [118].This limits the usage of RFA in practice, primarily due to the high chance of post-interventional complications. Furthermore, the clinical and technical challenges like insufficient ablation of tumors due to constraints of ablation needles, cooling of tissue by the adjacent blood vessels, large tumor mass, and tumors in the surroundings of heat-sensitive organs adds further complexity to the RFA procedure [118]. Nevertheless, we think that RFA will soon promote its clinical standing in treating advanced-stage liver tumors, primarily because of its potential to be used with multi-model imaging modalities.

The tumor location and the affected liver segment determine the nature of the radiological intervention and the prescribed segmentation method. It is recommended to use graph cut and gradient vector flow methods instead of active contour segmentation when the tumor is near the surface. This is due to the fact that the active contours can easily stream into the neighboring organs and cause over-segmentation, while gradient vector flow methods have demonstrated effective performance even for broken edges and subjective contours [21]. Furthermore, the liver segment location is vital in radiological interventions, because it can affect the dose limit in treatment planning [105]. For example, if the tumor is located in the caudate lobe, necessary safety precautions should be taken for positioning accuracy and quality assurance to avoid harming the gastrointestinal track [105]. A high degree of accuracy in target delineation and the use of image-guided radiotherapy (IGRT) can provide tighter margins that will minimize induced toxicity. Therefore, it is also recommended to segment the patient’s liver according to the Couinaud classification to recognize the risks associated with every segment.

Recurrence of HCC after resection is a frequent postoperative occurrence [119]. The literature shows that there is a difference in opinion among clinicians on the precise chance for recurrence after transplant or partial resection. Some works claim that over 50-80% of patients following resection develop recurrences (over the first two years) [120, 121], while others claim that only over 20% of patients are at the risk of developing tumor relapse [119]. Nevertheless, studies emphasize mandatory postoperative surveillance and regular screening [122]. Several key clinical indicators (like the presence of microscopic venous invasion, slow growth of small and inactive tumors) are signs of HCC recurrence [123]. It may be noted that the detection of tumor relapse also relies heavily on the postoperative imaging modality and segmentation tools. Thus, accurate and precise segmentation tools are required to detect minute lesions for early diagnosis of recurrent HCC. Models proposed by Alirr et al. [81], Wang et al. [79], and Vorontsov et al. [83] are well-suited for this task. Moreover, a combination of precise segmentation models with other clinical treatment methods like nucleic acid analogs and interferon (IFN) [124] can potentially become a robust curative option for HCC.

Though RFA, liver resection, and transplantation are effective remedying measures for treating HCC and most other hepatic diseases, they are not always used in isolation. Depending on the patient’s clinical history and pathological context, clinicians sometimes may need to use a combination of surgical and radiological interventions to achieve optimal results. Targeting numerous pathways in the HCC cascade with a variety of treatments can help in accomplishing personalized care aimed to improve overall survival [125]. Clinicians hope that combination therapies would have higher treatment efficacy and efficiency. Some promising compound treatment methods blend direct cytotoxicity from chemotherapeutic agents and ischemia from selective embolization to cause tumor necrosis. In addition, embolization results in reducing washout and systemic chemotherapy toxicity [126].

In addition to the patient’s clinical history, the diagnosis of HCC also relies on multiple technical (such as the choice of contrast, imaging modality, etc.) and pathological (liver heterogeneity, liver diseases, tumor size, and intensity) factors [22–24]. Due to this, the optimum course of treatment depends on many contextual factors and differs from patient to patient. Currently, well-established clinical guidelines and protocols attempt to account for these contextual factors to assist treatment decisions. They oversee the detection of small lesions and the classification of benign (regenerative nodule) or pre-malignant nodules from HCC [25].

### Limitations

This work aims to supplement the well-established clinical guidelines by suggesting varying segmentation methods appropriate for assisting diagnostic and therapeutic decision-making. However, we believe that our work still needs improvement: firstly, in order to manage the scope of our work, we have made the assumption that the segmentation methods are well-established and validated. Thus, we do not assess the pros and cons of each of the segmentation methods discussed, and focus instead on proposing a categorization schema of these segmentation methods with respect to their clinical utility. Secondly, in order to limit the scope of our work, we have not tried to address the existing challenges for the discussed segmentation methods and imaging modalities. It is expected that each segmentation method in combination with the imaging modality will have unique and patient-specific challenges due to heterogeneity in liver morphology and pathology. These challenges would need to be tackled as part of future work focusing on each segmentation method and procedure. Nevertheless, in this work, we have strived to establish a knowledge base of segmentation methods as an adjunct to an existing, well-established clinical decision-making process (i.e., outcomes of clinical protocols), thereby expediting the segmentation tool selection for treatment of HCC.

In future, we aim to assess the pros and cons of different segmentation methods for surgical and radiological interventions. Based on our assessment, we will design segmentation methods to overcome the limitations of existing methods and imaging modalities. Furthermore, we will explore segmentation methods for liver sub-segmentation to improve the success rate of radiological interventions preventing any harm to the healthy portions of the liver. Finally, we aim to evaluate the effectiveness of combined surgical and radiological interventions.

## Conclusion

This paper reviews state-of-the-art segmentation methods and categorizes them into three types of clinical intervention based on well-established clinical guidelines: transplantation, partial resection, and radiological interventions. This categorization is based on critical technical requirements or expectations from the algorithm to provide the best possible segmentation needed by the surgeon for a specific type of intervention. The broader aim is to contribute to optimal post-interventional outcome by aiding and supplementing the well-established yet complicated clinical diagnostic and therapeutic protocols. Considering each application (preoperative planning or image-guided intervention) have their own requirements in terms of accuracy and automation, we have also summarized the methods matching to their most appropriate corresponding clinical applications and types of intervention. We have observed that no single algorithm provides a ’one-size-fits-all’ solution. Therefore, we believe that our work could help the clinicians in choosing the appropriate algorithms on a case-by-case basis, ensuring optimized healthcare outcomes.

## Data Availability

All data generated or analysed during this study are included in this published article.

## Abbreviations

MRI: Magnetic resonance imaging
CT: Computed tomography
WHO: World Health Organization
HCC: Hepatocellular carcinoma
CAD: Computer-aided diagnoses
RECIST: Response Evaluation Criteria in Solid Tumor
RFA: Radiofrequency Ablation
PEI: Percutaneous Ethanol Injection
TACE: Transcatheter Arterial Chemoembolization
TNM: Tumor, Node, Metastasis
BCLC: Barcelona staging classification
CLIP: Cancer of the Liver Italian Program
CLMs: Colorectal Liver Metastases
DSC: Dice Similarity Coefficient
CNN: Convolutional neural network
VOE: Volumetric overlap error
LiTS: Liver Tumor Segmentation
MCG: Multi-scale candidate generation method
ACM: Active contour model
PNN: Probabilistic neural network
HFCNN: Hybridized Fully Convolutional Neural Network
mU-Net: Modified U-Net
AHC: Attention Hybrid Connection
ASSD: Average symmetric surface distance
SBRT: Stereotactic Body Radiation Therapy
TARE: Transarterial Radioembolization
PVE: Portal vein embolization
MWA: Microwave thermal ablation
FLR: Future Liver Remnant
TLV: Total Liver Volume
AMEM: Adaptive mesh expansion model
RMSE: Root mean square error
IGRT: Image-guided radiotherapy
IFN: Interferon

## Footnotes

1 https://flare.grand-challenge.org/.

2 https://kits21.kits-challenge.org/.
