# Correction: Practical utility of liver segmentation methods in clinical surgeries and interventions

**DOI:** 10.1186/s12880-022-00869-4

**Published:** 2022-08-08

**Authors:** Mohammed Yusuf Ansari, Alhusain Abdalla, Mohammed Yaqoob Ansari, Mohammed Ishaq Ansari, Byanne Malluhi, Snigdha Mohanty, Subhashree Mishra, Sudhansu Sekhar Singh, Julien Abinahed, Abdulla Al-Ansari, Shidin Balakrishnan, Sarada Prasad Dakua

**Affiliations:** 1 Surgical Research, Hamad Medical Corporation, Doha, Qatar; 2 Texas A&M University, Doha, Qatar; 3 McGill University, Montreal, Canada; 4 KIIT University, Bhubaneswar, India

## Correction to: BMC Medical Imaging (2022) 22:97 10.1186/s12880-022-00825-2

Following the publication of the original article [1] the authors requested to remove the statement "The Open Access funding was provided by Qatar National Library" from the "Funding" section of their article, as this is no longer applicable.
